# Comparative Distributional Impact of Routine Immunization and Supplementary Immunization Activities in Delivery of Measles Vaccine in Low- and Middle-Income Countries

**DOI:** 10.1016/j.jval.2020.03.012

**Published:** 2020-06-12

**Authors:** Allison Portnoy, Mark Jit, Stéphane Helleringer, Stéphane Verguet

**Affiliations:** Center for Health Decision Science (Portnoy), Department of Global Health and Population (Verguet), Harvard T.H. Chan School of Public Health, Boston, MA, USA; Department of Infectious Disease Epidemiology, London School of Hygiene and Tropical Medicine, London, England, UK (Jit); Modelling and Economics Unit, Public Health England, London, England, UK (Jit); Department of Population Family and Reproductive Health, Johns Hopkins Bloomberg School of Public Health, Baltimore, MD, USA (Helleringer).

**Keywords:** equity, low- and middle-income countries, measles, supplementary immunization activities, vaccination

## Abstract

**Objectives:**

In many countries, measles disproportionately affects poorer households. To achieve equitable delivery, national immunization programs can use 2 main delivery platforms: routine immunization and supplementary immunization activities (SIAs). The objective of this article is to use data concerning measles vaccination coverage delivered via routine and SIA strategies to make inferences about the associated equity impact.

**Methods:**

We relied on Demographic and Health Survey and Multiple Indicator Cluster Surveys multi-country survey data to conduct a comparative analysis of routine and SIA measles vaccination status of children by wealth quintile. We estimated the value of the angle, *θ*, for the ratio of the difference between coverage levels of adjacent wealth quintiles by using the arc-tangent formula. For each country/year observation, we averaged the *θ* estimates into one summary measurement, defined as the “equity impact number.”

**Results:**

Across 20 countries, the equity impact number summarized across wealth quintiles was greater (and hence less equitable) for routine delivery than for SIAs in the survey rounds (years) during, before, and after an SIA about 65% of the time. The equity impact numbers for routine measles vaccination averaged across wealth quintiles were usually greater than for SIA measles vaccination across country-year observations.

**Conclusions:**

This analysis examined how different measles vaccine delivery platforms can affect equity. It can serve to elucidate the impact of immunization and public health programs in terms of comparing horizontal to vertical delivery efforts and in reducing health inequalities in global and country-level decision-making.

## Introduction

Underlying differences in the social determinants of health create systematic differences in health among groups in society.^1^ In particular, health largely improves with increasing income^2–4^ for many reasons including differential access to health services according to the opportunities afforded by wealth, especially in low- and middle-income countries (LMICs).^5,6^ Without considering whom an intervention will reach and who can benefit most from the intervention, delivering public health programs and technologies to mitigate disease burden can further exacerbate these inequalities, as seen with unequal ownership of insecticide-treated nets for malaria control, for example.^7^ Although evidence shows that inequalities in under-5 mortality in LMICs are decreasing, large disparities still persist and highlight the need to prioritize inequality reduction and equity in decision-making at the global and national levels.^8^ Despite previous progress toward measles elimination and control efforts, the measles incidence has increased in 5 of 6 World Health Organization (WHO) regions since 2016, with reported cases increasing by 45% in LMICs receiving vaccination support from Gavi, the Vaccine Alliance.^9^ The burden of measles mortality is highest among vulnerable populations, including younger children (less than 5 years of age) and low-income countries, particularly in sub-Saharan Africa.^10,11^

Vaccines are one of the most effective public health interventions but need to reach all socioeconomic groups for maximal impact as well as for equity considerations.^12,13^ Nevertheless, routine coverage of measles-containing vaccine (MCV) varies substantially, with vaccine coverage up to 4 times higher in the wealthiest quintiles compared with the poorest quintiles.^13–16^ Achieving vaccination coverage equitably is an important consideration for national programs, not only because of the stated policy priorities of equity in health^17,18^ but also owing to the often higher disease burden in poorer compared with richer households in LMICs.^14^ National immunization programs can use 2 main modes of vaccine delivery: routine immunization and mass immunization campaigns or supplementary immunization activities (SIAs). To address equity considerations in vaccination programs, decision-makers need to weigh the costs and benefits of each delivery platform to determine the appropriate mix of services to achieve health and equity impact goals.

With routine immunization programs, vaccines are delivered at fixed sites (typically health facilities) on a consistent schedule, with vaccines in LMICs typically being made available periodically (daily, weekly, or monthly). On the other hand, the SIA strategy differs from routine vaccination in that the scheduling can often be determined by disease burden, the need to respond to potential outbreaks, and/or programmatic coverage needs, as well as global and regional control and elimination goals. In LMICs, SIAs are typically used to achieve specific goals, such as catching up people who were missed by routine immunization or achieving measles elimination or polio eradication.^19^ During a mass vaccination campaign or SIA, health workers and volunteers establish additional outreach service points (for measles vaccination) or go door to door (for polio vaccination) to offer immunizations to all members of a target population, irrespective of previous vaccination status.^19,20^ Because SIAs are more “vertical” in nature and require a level of surge capacity in terms of human and financial resources for vaccine delivery, they may present less consistency in terms of budgeting and allocation of healthcare workers. In addition, although a routine strategy can sometimes strengthen the capacity of the health system and be more “horizontal” in nature, SIAs may not strengthen general capacity directly, although they can contribute to the development of the health system in other ways, notably by reducing coverage disparities, reducing health inequalities and improving equity, and allowing other interventions to be delivered at the same time.^21,22^

The WHO recommends 2 doses of MCV, with the first dose administered at 9 to 12 months of age and the second dose at 15 to 18 months of age.^23^ Measles SIAs have been shown to improve equity in LMICs by strengthening coverage among children from lower socioeconomic status compared with the routine first dose of measles-containing vaccine (MCV1) delivered in the routine immunization program (eg, Expanded Programme on Immunization).^19,20,22^ The objective of this article is to use the Demographic and Health Survey (DHS) and Multiple Indicator Cluster Surveys (MICS) data concerning measles vaccination coverage delivered via SIA and routine MCV1 strategies to make inferences about the differential vaccine coverage impact across socioeconomic groups of such distinct delivery modes for immunization.

## Methods

### Data Extraction

The analysis focused on LMICs (as classified by 2019 World Bank income levels^24^) for which years and dates of measles SIAs were available from the WHO.^25^ The DHS and MICS surveys were selected from the available survey years that occurred 1 to 2 years after measles SIAs, after reviewing the schedule of SIAs in the identified countries from 2000 to 2014.^25–27^ The DHS and MICS data were included according to availability of the “vaccinated during campaign” indicator, to determine if SIA (campaign) vaccination status was included in addition to MCV1 status during the administration of the DHS or MICS survey. In the included surveys, mothers were asked whether their children participated in a specific SIA (with possible answers being “yes,” “no,” or “don’t know”), for which the date of implementation was available.^25^ We then extracted data for routine vaccination status across several years, or “rounds,” of both the DHS and MICS. For each available survey round during the same year as an SIA or 1 to 2 years after an SIA, where available, we also obtained routine vaccination status for the same survey round (“MCV1 During”), the round immediately before the SIA round (“MCV1 Before”), the round immediately after the SIA round (“MCV1 After”), the round that occurred 2 rounds before the SIA round, and the round that occurred 2 rounds after the SIA round.

We relied on DHS or MICS data to determine the routine and SIA vaccination status of children. The DHS and MICS are nationally representative household-based surveys conducted periodically in LMICs using a well-established standardized sampling frame and methodology.^26–29^ The first round of DHS began in 1984 and the first round of MICS in 1995. For each survey program, more than 300 surveys have been conducted in more than 90 countries. A mix of survey tools including both household and individual questionnaires are conducted according to a 2-stage cluster design under programs developed by ICF International (DHS) and UNICEF (MICS).^26,27^ Each country survey includes a vaccination history for surviving children who are younger than 5 years at the time of the survey. The interviewing approach of these surveys, which reconstruct the child’s history of vaccination according to the child’s health card and/or maternal reports of prior vaccination, is currently the best practice to determine the proportion of children covered by each vaccine at the time of the survey.^30^ Specifically, for routine vaccination, if the health card of the child is available, interviewers ask to see the card and transcribe the dates of each vaccination recorded on the card and also ask if the child has obtained other vaccinations that are not recorded. If the card is not available, interviewers ask the mother/guardian whether the child has received doses of each vaccine at any time before the survey and, if so, how many doses.^19,20^

We relied on the selected surveys where specific questions about SIAs were asked to classify SIA vaccination. Data included both measles routine vaccination status as described above and SIA vaccination status, child age at time of vaccination, and household wealth quintile from the unweighted sample of DHS or MICS data. Household wealth quintile, according to the wealth index based on ownership of selected assets, is defined as poorest, poorer, middle, richer, and richest.^26^ All data were extracted by a single author.

### Analysis

Using the extracted data for the various DHS or MICS rounds, we conducted a comparative, quantitative analysis of measles vaccination coverage and equity at the country level. First, we estimated the measles vaccination coverage of each DHS or MICS by household wealth quintile as the number of children reached by either measles SIA or measles routine immunization program for that wealth quintile divided by the total size of the population in that wealth quintile.

**Figure 1.**
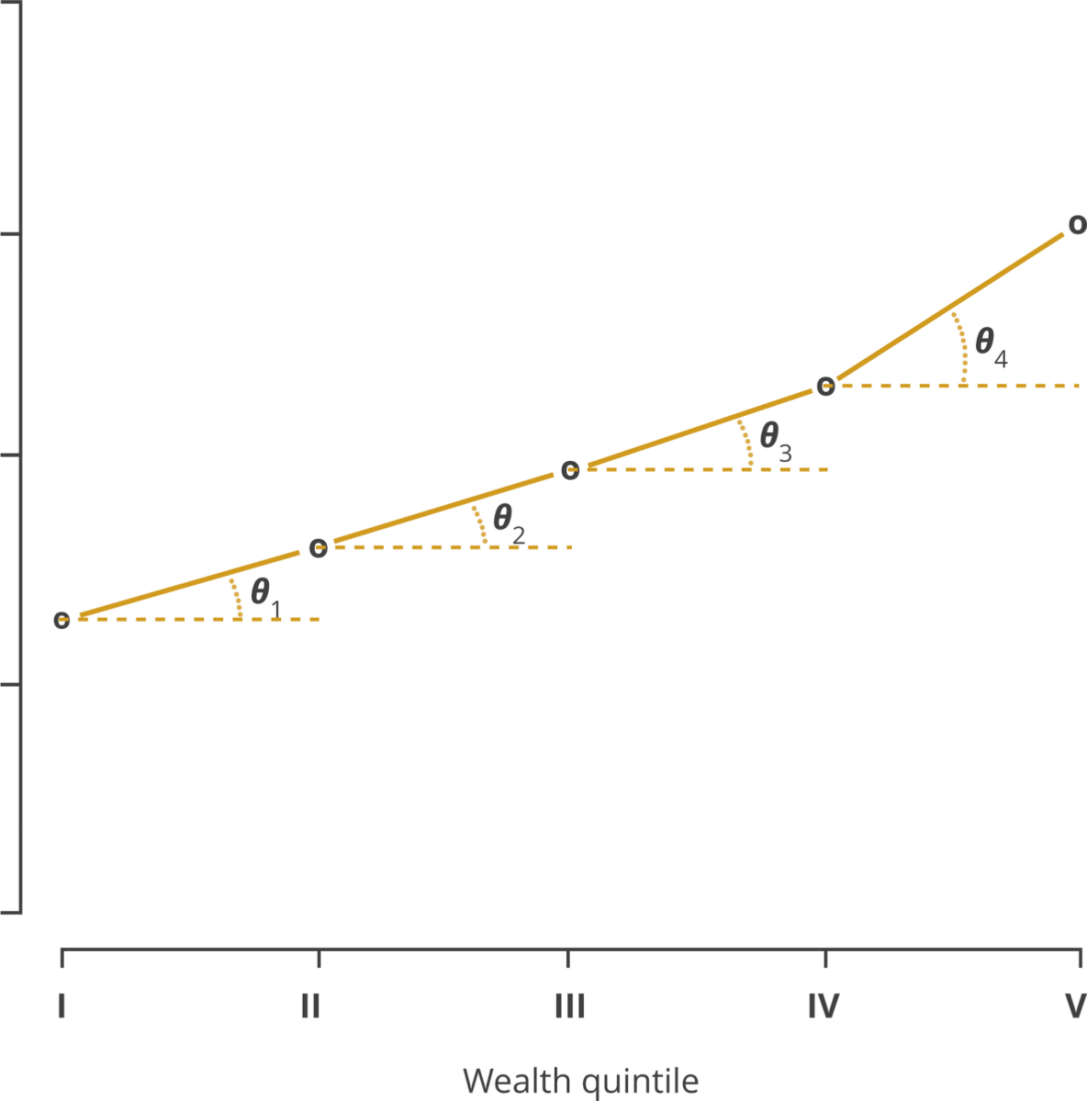
The angle, *θ*, capturing the coverage-level differences between adjacent wealth quintiles. This illustrative example relies on data from the supplementary immunization activities (SIA) in Ghana from the 2008 Demographic and Healthy Survey (DHS).

Second, we estimated the value of the angle, denoted *θ* (in degrees), for the ratio of the difference between the coverage levels of adjacent wealth quintiles (and the difference between adjacent wealth quintiles, ie, interpreted quantitatively as one) by using the arc-tangent formula (Fig. 1). As the value of *θ* increases, the distribution of vaccination across wealth quintiles becomes less equitable. The equation for *θ* is given by

(1) $$\tan\left(\theta_{i}\right)=\frac{180}{\pi }*\frac{\;Coverage{\;}_{i+1}-\;Coverage{\;}_{i}}{\;Wealth\;Quintile{\;}_{i+1}-\;Wealth\;Quintile{\;}_{i}},\;i\in 1:4$$

The multiplier $\frac{180}{\pi }$ was used to obtain angles measured in degrees. We adjusted the value of *θ* by a factor of 4 because of the 4 categories of adjacent wealth quintile differences (ie, richest vs rich, rich vs middle, middle vs poor, and poor vs poorest) in order to maintain an orthometric system of coordinates to derive a tangent estimate. A zero value of *θ* indicates that coverage between adjacent quintiles was equal (including equal to zero). A negative value of *θ* indicates that coverage actually improved for poorer quintiles. As the minimum possible coverage level is 0% and the maximum is 100%, the numerator varies between the extreme values of –100 and 100 such that *θ* can vary between –90° and +90°. For each country/year observation, we then averaged the 4 *θ* estimates into 1 summary *θ* measurement, which we called the “equity impact number.” We also calculated an independent 2-sample *t* test for the equity impact numbers for routine measles vaccination from each DHS round compared with the equity impact numbers for measles SIA vaccination.

We considered 2 sensitivity analyses for comparisons with the equity impact number. First, we compared the poorest quintile to the richest quintile and analyzed the rudimentary *θ* extracted from this direct comparison. Second, we conducted pairwise comparisons across all quintiles and calculated a summary *θ* by averaging over all comparisons.

## Results

After examination of the DHS and MICS data, 20 countries and 22 survey-years were identified with the necessary SIA information. The final 20 countries included in the analysis had surveys ranging from 2002 to 2014 for measles SIAs. The details of the DHS and MICS included in the analyses are provided in Table 1, as well as contextual information such as gross domestic product per capita to indicate financing capacity^24^ and under-5 population to indicate routine target population size.^31^

**Figure 2.**
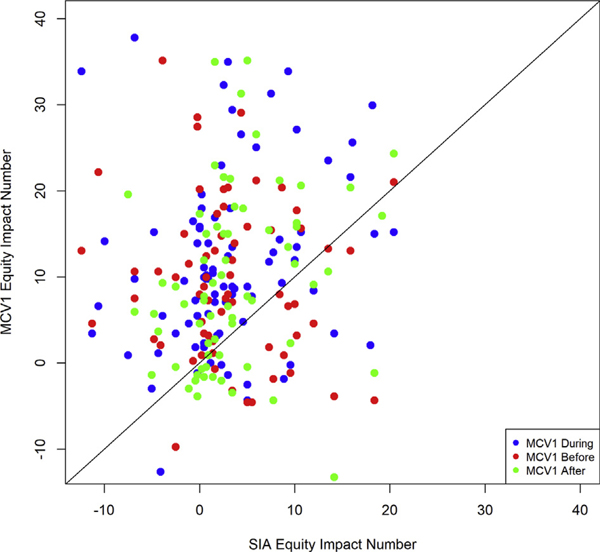
The routine measles vaccination equity impact number *θ* compared with the SIA measles vaccination equity impact number *θ* for different wealth quintiles. After indicates MCV1 from the survey round immediately after the SIA round; Before, MCV1 from the survey round immediately before the SIA round; During, MCV1 from the survey round that also collected SIA coverage; MCV1, routine measles-containing vaccine, first dose; SIA, supplementary immunization activities. A point located above the diagonal line indicates MCV1 *θ* being greater than SIA *θ*. Each point represents a pairwise relationship between adjacent wealth quintiles (eg, pair of coverage for quintile II and coverage of quintile III).

The MCV1 equity impact number *θ* in the survey round during the year(s) of the SIA ranged from 1.1° to 25.5°, whereas the SIA *θ* ranged from –7.6° to 9.8° (Table 2). Overall, the routine MCV1 *θ* in the survey round during the year(s) of the SIA was greater than the SIA *θ*, with an average of 11.4° (standard deviation [SD]: 6.2°) compared with 3.1° (SD: 3.7°). Across all 20 countries, the MCV1 *θ* across wealth quintiles was greater than the SIA *θ* in the survey rounds during, before, and after the SIA approximately 65% of the time. In addition, for survey rounds during, before, and after the SIA, if the country with the largest equity impact number for MCV1 (Nigeria) were removed, the mean values in Table 2 would decrease (become more equitable) but still remain greater than the mean SIA *θ*. According to an independent 2-sample *t* test, the MCV1 *θ* for the same survey round as the SIA was greater than the SIA *θ* (*P* < .001), the MCV1 *θ* for the previous survey round to the SIA was greater than the SIA *θ* (*P* = .011), and the MCV1 *θ* for the subsequent survey round to the SIA was greater than the SIA *θ* (*P* < .001). For all pairwise relationships of wealth quintiles (eg, pair of coverage for quintile II and coverage of quintile III), the likelihood of the MCV1 equity impact number *θ* being greater than the SIA measles vaccination *θ* is shown in Figure 2.

**Figure 3.**
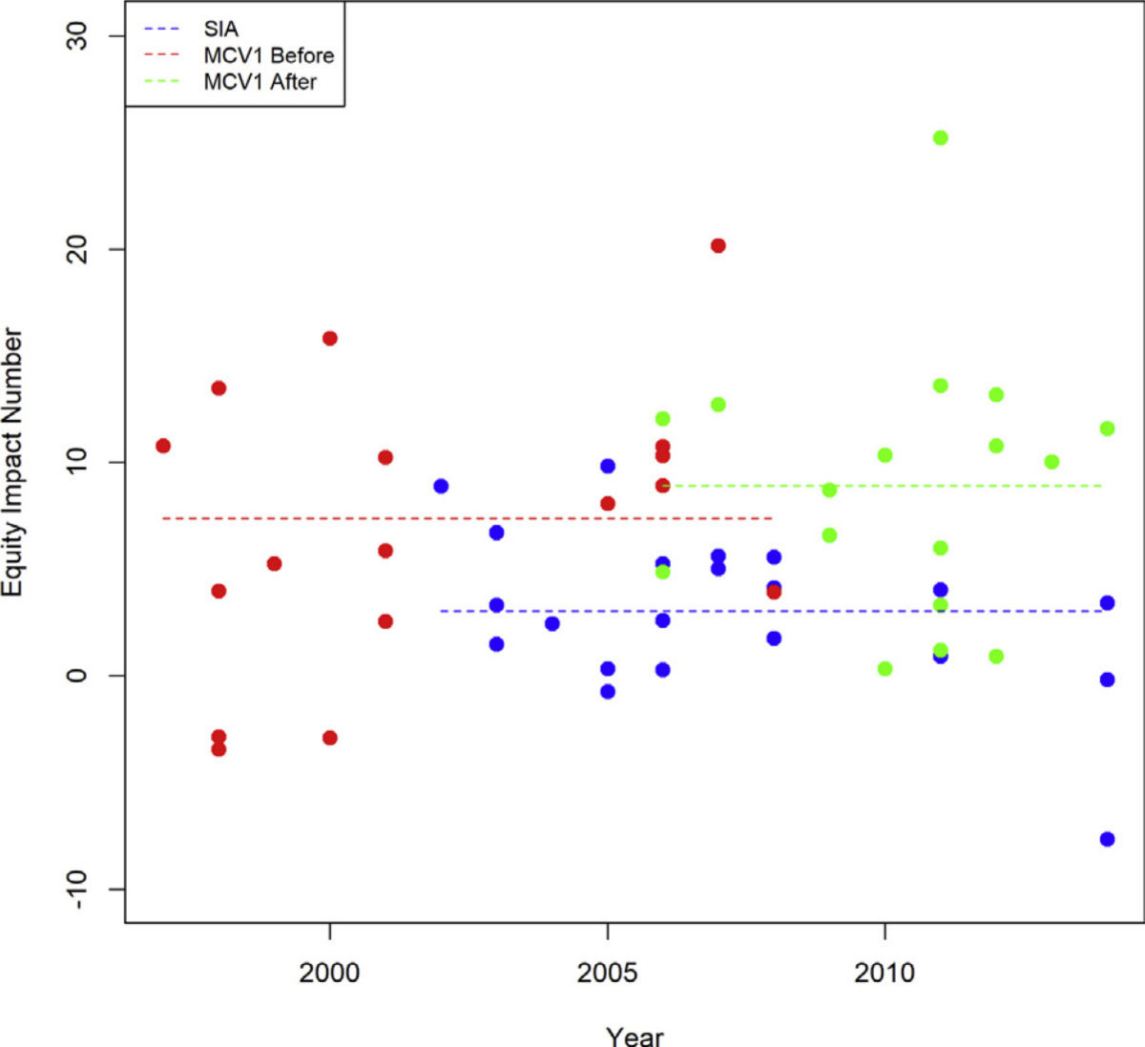
Equity impact numbers *θ* for routine and SIA measles vaccination over time and on average. The dots represent the equity impact numbers by country, and each dashed line represents the average equity impact number by delivery platform (routine vs SIA measles vaccination). After indicates MCV1 from the survey round immediately after the SIA round; Before, MCV1 from the survey round immediately before the SIA round; DHS, Demographic Health Survey; MCV1, routine measles-containing vaccine, first dose; SIA, supplementary immunization activities.

The equity impact numbers *θ* for routine measles vaccination averaged across wealth quintiles were greater on average than the *θ* numbers for SIA measles vaccination. This relationship was consistent over time and was maintained whether the MCV1 dose was given before or after an SIA round (Fig. 3).

In sensitivity analyses, a comparison between only the poorest quintile and the richest quintile did not change the relationship of the summary *θ* between MCV1 and SIA vaccination. According to an independent 2-sample *t* test, this specific pairwise relationship was significant at the 5% level for the MCV1 *θ* for the same (*P* < .001), previous (*P* = .03), and subsequent (*P* = .001) survey round compared with the SIA *θ*. The MCV1 *θ* in the survey round during the year(s) of the SIA ranged from 4.6° to 62.5°, whereas the SIA *θ* ranged from –28.4° to 34.8°. Overall, the MCV1 *θ* in the DHS round during the year(s) of the SIA was still greater than the SIA *θ* in approximately 91% of country-year observations at an average of 37.1° (SD 15.5°) as compared with 11.8° (SD 13.6°).

When all pairwise comparisons between each of the 5 quintiles are considered, the MCV1 *θ* in the survey round during the year(s) of the SIA was greater than the SIA *θ* in approximately 81% of country-year observations at an average of 13.6° (SD 14.5°) as compared with 3.8° (SD 8.3°). This relationship was also statistically significant (*P* < .001).

## Discussion

Consistent with previously published studies,^19,22^ our analysis found that measles SIAs tend to provide a more equal coverage across socioeconomic groups than routine measles immunization programs. It is likely that countries with relatively low coverage for measles in the routine vaccination program, such as Nigeria, might also be less equitable in that coverage, indicating a possible correlation between the MCV1 *θ* and the strength of the routine immunization program. In fact, Nigeria had the lowest MCV1 coverage (43%) of the countries in our study, around the years of its SIA, and the largest discrepancy between *θ* for MCV1 and SIA.^15^ Nevertheless, there may be additional costs associated with relying on this type of vertical delivery platform. SIAs are a vital complement to routine immunization programs that are intended to close the coverage gaps left by incomplete access to such programs, but they may still be unable to reach children not previously reached by routine programs in LMICs.^20^ In addition, there may be opportunity costs (eg, diversion of human resources’ and health workers’ time) associated with SIAs that can negatively affect the functioning of health systems, for example, by potentially reducing care seeking and use of select routine child and maternal health services during SIA rollout.^32,33^ Nevertheless, the evidence on how SIAs are affecting the use of routine health services remains mixed.^33^ All of these benefits and costs of SIAs on routine immunization and routine services likewise have implications for the impact of vertical delivery platforms on health system strengthening.^21,34^ Increasing the equity of healthcare delivery in LMICs requires balancing the choice of targeted programs versus broad universal coverage. In this respect, the equity impact number, *θ*, can serve as a simple intuitive measure to summarize the differential equity impact of distinct health system delivery platforms and the mixed use of targeted versus universal public health programs. Therefore, it may also serve to summarize the equitable coverage of other public health interventions.

There are several limitations to this analysis. First, the small sample size in the numbers of children with measles SIA coverage data (less than 10 000 children per country across 20 LMICs) collected in the DHS and MICS is a limiting factor. Second, because SIA data were derived from the DHS or MICS question on whether a “child was vaccinated during campaign,” there is uncertainty regarding the accuracy of the reported information, as it is subject to mothers’ reporting and recall biases. In addition, the DHS and MICS data use complex sampling and require weights for country-level estimates, but this analysis was unweighted, and estimates thus do not indicate national-level vaccination coverage. Third, we have not addressed the differential impact that MCV1 and SIA vaccine delivery may have according to individual-level vaccination status. In other words, a child reached by both MCV1 and SIA would gain less from the SIA dose, in terms of vaccine efficacy and protection, than a child receiving her or his first dose of MCV from the SIA because she or he missed the routine visit. A previously published analysis addressed this concern,^20^ and we have chosen here to focus our analysis on the differential coverage implications by vaccine delivery platform type. Likewise, we were not able to compare the differential impact between the routine second dose of MCV (MCV2) and SIA delivery, as the DHS and MICS data analyzed did not include MCV2 coverage. In addition, only 3 countries included in the analysis had introduced MCV2 at the time of their analyzed SIA. Fourth, as SIAs offer immunizations to all members of a target population, irrespective of previous vaccination status,^19,20^ we assumed that there was no systematic impact on the coverage levels of the routine program after SIAs. Nevertheless, it may be the case that individuals reached by SIAs are not subsequently recorded as routinely vaccinated in DHS or MICS survey rounds. We attempted to address this limitation by analyzing as many survey rounds and countries that had the relevant data available. There may likewise be exogenous factors that influence coverage levels of programs over time, which we have not accounted for in this analysis. Nevertheless, when examining the changes in MCV1 coverage between SIA years and survey years, we found that coverage changed by 3.4 percentage points (5% change) on average across the analyzed sample.^15,25^ Finally, we opted for a simple metric, easily interpretable and replicable, of vaccine coverage disparities, whereas other more sophisticated inequality measurement approaches, such as concentration curves and indices,^35^ could also be used. The equity impact number (*θ*), which we introduced in this article, has the advantage of providing an intuitive geometrical interpretation in directly assessing the distance between 2 groups in terms of both wealth and coverage. Compared with only the ratio or difference between the poorest and richest quintiles, *θ* can be calculated as a summary measure for all pairwise comparisons across wealth quintiles in a population. This measure can therefore address the entire socioeconomic distribution, similar to more technical wealth-based measures such as the slope index of inequality (for absolute inequality) and the concentration index (for relative inequality).^36–38^ Our proposed measure is therefore both simple to calculate and provides detailed distributional information, and it could be used as a first step in decision-making processes as a means to identify when further evaluation is warranted.

In summary, this analysis examined how different measles vaccine delivery programs can have a differential impact on the equity implications of immunization programs. This can enable better description of the real-world impact of different delivery platforms in reducing health inequalities and improving equity at the global and local levels and can further highlight the important role that measles SIAs can play in reaching children from poorer households. SIAs serve as a complement to routine programs by periodically offering immunizations to all members of a target population, irrespective of previous vaccination status,^19,20^ especially where health systems are weak and vaccination coverage is low, as recommended by the WHO.^23^ High coverage of measles vaccine (95%) across all socioeconomic groups is essential to achieve the herd immunity levels necessary for measles elimination.^23^ With an improved estimation of public health impact, determining the mix of routine and SIA delivery necessary for measles control and elimination efforts can help achieve not only the necessary coverage levels for preventing measles cases and deaths but also those levels equitably across socioeconomic groups. Although measles SIAs have been shown to be cost-effective, the cost and cost-effectiveness will vary by setting and may be improved with integrated delivery of multiple interventions.^39,40^ Equity considerations can complement the elements of cost, cost-effectiveness, and feasibility that decision-makers may use to decide the appropriate mix of delivery platforms for measles vaccine. Nevertheless, decision-makers will also need to weigh this important information against health systems’ strengthening considerations when considering how to address disease prevention and control, beyond the sole case study of measles vaccine delivery here.

## Conclusions

We studied the differential coverage impact of MCV1 versus SIA delivery of measles vaccine in defining and comparing an equity impact number across the 2 distinct vaccine delivery modes. Across 20 LMICs, we found that the likelihood that the MCV1 equity impact number was greater (hence more inequal) than the SIA equity impact number was about 65% of the time (at the 5% significance level). We also found that, when examining the trends across time, the equity impact numbers for MCV1 measles vaccination averaged across wealth quintiles were greater on average than the equity impact numbers for SIA measles vaccination. The similar levels in the equity impact numbers before and after measles SIAs indicated that there may be a systematic difference in the current distributional implications across these two distinct modes of vaccine delivery.

## Supplementary Material

1

## Figures and Tables

**Table 1.** Details of measles SIAs and survey year(s) for DHS and MICS, by country.

Country	WHO region	GDP per capita (2018 USD)^17^	Under-5 population (2018)^22^	SIA year(s) and target population^18^	DHS or MICS survey year(s)*

Benin	AFR	$900	1 910 000	2005: 1 058 201	2001, **2006,** 2011
Burkina Faso	AFR	$720	3 470 000	2001: 5 139 696	1993, 1998, **2003,** 2010
Cameroon	AFR	$1530	4 120 000	2012: 3 507 987	2006, **2014**
Democratic Republic of the Congo	AFR	$560	15 800 000	2007: 3 736 672	2001, **2007,** 2010, 2013
Ghana	AFR	$2200	4 170 000	2001: 801 694	1993, 1998, **2003,** 2006, **2008,** 2011, 2014
				2002: 7 673 593	
				2006: 5 065 661	
Guinea	AFR	$880	2 100 000	2002: 789 203	1999, **2005,** 2012
				2003: 3 278 577	
Guinea-Bissau	AFR	$740	305 000	2012: 247 786	2006, **2014**
Haiti	AMR	$870	1 260 000	2004: 799 325	2000, **2005,** 2012
Honduras	AMR	$2500	1 020 000	2004: 759 794	**2005,** 2011
Indonesia	SEAR	$3890	23 700 000	2002: 2 833 430	1997, **2002,** 2007, 2012
Iraq	EMR	$5830	5 380 000	2010: 2 794 889	**2011**
Kenya	AFR	$1710	7 040 000	2002: 13 582 031	1993, 1998, **2003,** 2009, 2014
Lesotho	AFR	$1300	254 000	2000: 624 994	2000, **2004,** 2009, 2014
				2003: 204 786	
Nepal	SEAR	$1030	2 710 000	2005: 4 326 348	1996, 2001, **2006,** 2011, 2014
Niger	AFR	$410	4 790 000	2004: 5 128 821	1998, **2006,** 2012
				2005: 325 281	
Niger	AFR	$2030	33 900 000	2005: 29 500 000	2003, 2007, **2008,** 2011, 2013
				2006: 31 630 011	
São Tomé and Príncipe	AFR	$2000	31 800	2007: 64 081	**2008, 2014**
				2012: 21 380	
Sierra Leone	AFR	$530	1 160 000	2003: 2 599 098	2005, **2008,** 2010, 2013
				2006: 748 209	
Vanuatu	WPR	$3120	42 100	2006: 79 063	**2007**
Vietnam	WPR	$2570	7 890 000	2010: 7 292 713	2006, **2011,** 2013

AFR indicates WHO African Region; AMR, WHO region of the Americas; DHS, Demographic and Health Survey; EMR, WHO Eastern Mediterranean region; GDP, gross domestic product; MICS, Multiple Indicator Cluster Survey; SEAR, WHO Southeast Asian region; SIAs, supplementary immunization activities; WHO, World Health Organization; WPR, WHO Western Pacific region.

* DHS or MICS survey year during the year of the implementation of SIA is highlighted in bold. Sources: DHS,^18^ MICS,^19^ United Nations World Population Prospects,^22^ World Health Organization,^18^ World Bank.^17^ Only DHS and MICS data were used in this analysis, but United Nations, WHO, and World Bank indicators were obtained for descriptive context.

**Table 2.** Equity impact number (*θ*, in degrees) by country, year, and type (MCV1 vs SIA) of vaccine delivery.*

Country	SIA	MCV1					MCV1 Mean
		DHS/MICS 2 rounds before SIA	DHS/MICS round before SIA	DHS/MICS round during SIA	DHS/MICS round after SIA	DHS/MICS 2 rounds after SIA	
	(2002–2012)	(1993–2003)	(1997–2008)	(2002–2014)	(2006–2014)	(2008–2014)	

Benin	2.6	NA	5.9	15	13.6	14.5	10.3
Burkina Faso	1.5	0.6	4	12.9	4.9	7.7	5.3
Cameroon	‒7.6	NA	10.8	23.7	NA	NA	8.9
Democratic Republic of the Congo	5.6	NA	2.6	18.8	10.4	12.8	10
Ghana (2003)	6.7	‒3.8	13.5	9.5	12.1	4.4	7.1
Ghana (2008)	5.6	9.5	12.1	4.4	3.3	2.7	6.3
Guinea	9.8	NA	5.3	15.1	13.2	NA	10.9
Guinea-Bissau	3.4	NA	10.3	10.6	NA	NA	8.1
Haiti	0.3	NA	15.8	9.6	0.9	NA	6.7
Honduras	‒0.7	NA	NA	1.1	1.2	NA	0.5
Indonesia	8.9	NA	10.8	11.3	12.7	12.1	11.2
Iraq	0.9	NA	NA	10.1	NA	NA	5.5
Kenya	3.3	2.2	‒3.4	11.7	6.6	7.9	4.7
Lesotho	2.5	NA	‒2.9	3.6	8.7	3.3	3.0
Nepal	0.3	4.7	10.2	11.5	6	8.6	6.9
Niger	5.3	NA	‒2.9	15.4	10.8	NA	7.2
Nigeria	4.0	20.4	20.2	25.5	25.2	27.7	20.5
São Tomé and Príncipe (2008)	4.2	NA	NA	3.9	11.6	NA	6.6
São Tomé and Príncipe (2014)	‒0.2	NA	3.9	11.6	NA	NA	5.1
Sierra Leone	1.8	NA	8.1	6.1	0.3	1.4	3.5
Vanuatu	5.0	NA	NA	14.3	NA	NA	9.7
Vietnam	4.0	NA	8.9	5.6	10.0	NA	7.2
Mean (SD)	3.1 (3.7)	5.6 (8.5)	7.4 (6.5)	11.4 (6.2)	8.9 (6.1)	9.4 (7.5)	7.5 (3.9)

DHS indicates Demographic Health Survey; MCV1, routine measles-containing vaccine, first dose; MICS, Multiple Indicator Cluster Surveys; NA, not applicable; SIA, supplementary immunization activities.

* Two different SIAs were included in the analysis for the countries of Ghana and São Tomé and Príncipe.
