# Supplementary material for: Comparative Distributional Impact of Routine Immunization and Supplementary Immunization Activities in Delivery of Measles Vaccine in Low- and Middle-Income Countries
Source: Value Health. Author manuscript; Available in PMC 2021 Jul 1. (PMC7519803; doi:10.1016/j.jval.2020.03.012)
Supplement: 1 [file NIHMS1589030-supplement-1.docx]

**Table S1. Details of surveyed sample from Demographic and Health Surveys and Multiple Indicator Cluster Surveys included in analysis***

| **Country** | **DHS or MICS survey year(s)*** | **Wealth Quintile** | | | | |
| --- | --- | --- | --- | --- | --- | --- |
|  |  | **Richest** | **Richer** | **Middle** | **Poorer** | **Poorest** |
| Benin | 2006 | 2,390 (15%) | 3,143 (20%) | 3,368 (21%) | 3,370 (21%) | 3,804 (23%) |
| Burkina Faso | 2003 | 1,729 (16%) | 1,916 (18%) | 2,774 (26%) | 2,251 (21%) | 1,975 (19%) |
| Cameroon | 2014 | 1,159 (16%) | 1,429 (20%) | 1,630 (23%) | 1,639 (24%) | 1,224 (17%) |
| Democratic Republic of the Congo | 2007 | 1,483 (16%) | 1,871 (21%) | 1,745 (19%) | 1,855 (21%) | 2,038 (23%) |
| Ghana | 2003 | 479 (12%) | 539 (14%) | 682 (18%) | 859 (22%) | 1,285 (34%) |
| Ghana | 2008 | 357 (12%) | 502 (17%) | 504 (17%) | 656 (22%) | 973 (32%) |
| Guinea | 2005 | 840 (15%) | 1,202 (22%) | 1,339 (26%) | 1,202 (22%) | 840 (15%) |
| Guinea-Bissau | 2014 | 609 (8%) | 1,076 (14%) | 1,720 (23%) | 1,864 (25%) | 2,304 (30%) |
| Haiti | 2005 | 694 (12%) | 1,091 (18%) | 1,262 (21%) | 1,290 (21%) | 1,678 (28%) |
| Honduras | 2005 | 1,149 (11%) | 1,510 (14%) | 1,855 (17%) | 2,648 (25%) | 3,638 (33%) |
| Indonesia | 2002 | 2,602 (16%) | 2,476 (15%) | 2,620 (16%) | 3,144 (19%) | 5,364 (34%) |
| Iraq | 2011 | 3,571 (10%) | 4,938 (14%) | 6,690 (18%) | 8,693 (24%) | 12,415 (34%) |
| Kenya | 2003 | 1,319 (22%) | 937 (16%) | 1,077 (18%) | 1,117 (19%) | 1,499 (25%) |
| Lesotho | 2004 | 584 (16%) | 636 (17%) | 652 (18%) | 943 (25%) | 882 (24%) |
| Nepal | 2006 | 846 (15%) | 1,012 (17%) | 1,044 (18%) | 1,212 (21%) | 1,669 (29%) |
| Niger | 2006 | 2,582 (28%) | 1,835 (20%) | 1,495 (16%) | 1,534 (17%) | 1,747 (19%) |
| Nigeria | 2008 | 3,808 (13%) | 4,755 (17%) | 5,609 (20%) | 6,871 (24%) | 7,604 (26%) |
| São Tomé and Príncipe | 2008 | 227 (12%) | 362 (19%) | 405 (21%) | 461 (24%) | 476 (24%) |
| São Tomé and Príncipe | 2014 | 248 (12%) | 375 (18%) | 404 (20%) | 462 (23%) | 541 (27%) |
| Sierra Leone | 2008 | 1,024 (18%) | 1,149 (20%) | 1,163 (21%) | 1,062 (19%) | 1,233 (22%) |
| Vanuatu | 2007 | 418 (26%) | 332 (20%) | 278 (17%) | 295 (18%) | 311 (19%) |
| Vietnam | 2011 | 775 (21%) | 737 (20%) | 649 (18%) | 595 (16%) | 922 (25%) |

*The surveyed sample includes the individuals with routine first dose of measles-containing vaccine (MCV1) and supplementary immunization activities (SIA) vaccination status and household wealth quintile defined. The proportion of the total sample size is included in parentheses. The survey years are the available survey years from the DHS and MICS that occurred one to two years following measles SIAs.
